# Transcatheter aortic valve replacement in patients with quadricuspid aortic valve in a single center

**DOI:** 10.3389/fcvm.2022.1011466

**Published:** 2022-09-28

**Authors:** Yang Liu, Mengen Zhai, Yu Mao, Chennian Xu, Yanyan Ma, Lanlan Li, Ping Jin, Jian Yang

**Affiliations:** Department of Cardiovascular Surgery, Xijing Hospital, Air Force Medical University, Xi’an, China

**Keywords:** quadricuspid aortic valve, aortic stenosis, aortic regurgitation, transcatheter aortic valve replacement, transcatheter

## Abstract

**Background:**

Quadricuspid aortic valve (QAV) is a rare congenital malformation that can present with aortic regurgitation or aortic stenosis (AS)), requiring surgical treatment. Transcatheter aortic valve replacement (TAVR) is an alternative treatment for older patients and its prognosis for QAV therapy remains challenging. We sought to examine our early experience with TAVR in patients with QAV.

**Materials and methods:**

Prospectively collected data were retrospectively reviewed in patients with QAV undergoing TAVR in our institution.

**Results:**

Five patients with QAV and AR or AS were treated with TAVR between January 2016 and January 2022. The mean age was 73.8 years (range 69–82 years), and the median Society of Thoracic Surgeons score was 7.51% (range 2.668–18.138%). Two patients had type B and three had either type A, D, or F according to the Hurwitz and Roberts classification for QAV. Four patients with pure aortic regurgitation underwent transapical TAVR using the J-Valve system, and the patient with severe AS underwent transfemoral TAVR using the Venus-A system. Procedural success was achieved in all five patients. Trivial paravalvular leak was only detected in one case after the procedure, and one patient received a permanent pacemaker due to high-degree atrioventricular block three days later. The median follow-up period was 18 (12–56) months. After discharge, no deaths occurred during the 1 year follow-up. All patients improved by ≥1 New York Heart Association functional class at 30 days; four patients were in functional class ≤II later in the follow-up period. All patients’ heart failure symptoms improved considerably.

**Conclusion:**

Our early experience with TAVR in QAV demonstrates these procedures to be feasible with acceptable early results. Further follow-up is necessary to determine the long-term outcomes of this modality.

**Clinical trial registration:**

[ClinicalTrials.gov], identifier [NCT02917980].

## Introduction

Quadricuspid aortic valve (QAV) is a rare congenital anomaly, with an incidence of 0.006 to 0.04% based on autopsy and echocardiography data (1, 2). Leonardo da Vinci first described the anatomical morphology of the QAV in his anatomical drawings more than 500 years ago (3). However, the characteristics, natural history, and specific recommendations for the management of patients with QAV are poorly defined because of its rarity. Limited studies and case reports have shown that more than half of patients with QAV will progress to the stage requiring surgery. Most patients also have progressive aortic valve regurgitation (AR), whereas aortic stenosis (AS) and ascending aortic enlargement are uncommon (4–6). Patients aged 50 to 70 years with aortic valve disease often require surgery. Surgical options for QAV include aortic valve repair and aortic valve replacement, but data on long-term clinical and surgical outcomes are lacking. Based on limited previous reports, the prognosis of surgery for QAV is equivalent to that of other aortic valve surgeries for younger patients with acceptable surgical risks (1, 4, 7).

Transcatheter aortic valve replacement (TAVR) has developed into a mature procedure over the past 20 years, and it has become the preferred least invasive treatment of choice for patients with high or inoperable surgical risks. The latest guidelines also extend the indications for TAVR to intermediate-risk or even to older low-risk patients (8, 9). A growing number of patients with aortic valve disease are treated with TAVR. It is now increasingly applied in different valvular malformations, including bicuspid aortic valves, native pure AR, valve-in-valve procedures for degenerated bioprostheses, and more complex anatomical characteristics. However, only a few cases of stenosed or regurgitant QAV treated successfully with TAVR have been reported (10–14). Our institution has more than 10 years of experience with TAVR and other structural heart interventions. Of more than 800 TAVR procedures, we performed five TAVR procedures for patients with QAV. Our goal was to present our early experiences with transapical and transfemoral TAVR for patients with QAV-related pure AR and severe stenosis. To our knowledge, this is the largest single-center cohort of patients with QAV treated with TAVR published to date.

## Materials and methods

### Patient population

**FIGURE 1:**
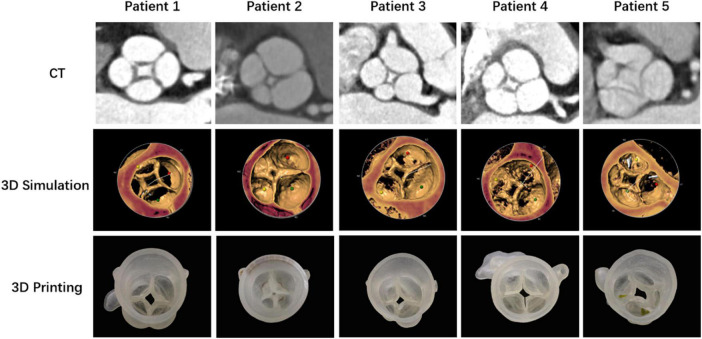
Anatomical structures of the quadricuspid aortic valves of five patients demonstrated with computed tomography angiography (CTA), 3-dimensional (3D) simulation, and 3D printing models.

All patients with a QAV have been identified, and underwent TAVR at the Xijing Hospital. Patients were diagnosed by transthoracic echocardiography before the procedure. After reviewing the patients’ ages, comorbidities, degrees of frailty, and moderate-to-high operative risk, the heart team recommended that all five undergo the TAVR procedure. Computed tomography angiography (CTA), performed routinely as part of the pre-TAVR work-up at our institution, revealed QAV with four separate leaflets in all patients. The dimensions of the aortic annulus, aortic root, left ventricle and left ventricular outflow tract were measured based on preoperative CTA images. 3-Dimensional (3D) printing models of the anatomical structure of the aortic root were reconstructed according to the preoperative CTA, which assisted the operator to more intuitively observe the anatomy of the valvular malformations and simulate the procedural plan (Figure 1).

Preoperative and postoperative data were collected prospectively and entered into the institutional database. All clinical files were reviewed, and perioperative characteristics were documented, including procedural time, fluoroscopic time, and postoperative hospital stay. All patients were seen in the clinic to ascertain their clinical status [New York Heart Association (NYHA) functional class] and adverse events after discharge. Transthoracic echocardiography was performed to evaluate the improvements in the construction and function of the patients’ hearts at 30 days, 6 months, 1 year, and yearly thereafter. CTA was also performed during the follow-up period in some patients. All patients or guardians of patients provided informed consent to participate in the study, and all clinical documents were reviewed for analysis.

### Hurwitz and Roberts classification

According to Hurwitz and Roberts classification, QAVs were classified into seven types: (a) QAV with four equal-sized cusps; (b) QAV with three equal-sized cusps and one smaller cusp; (c) QAV with two equal larger cusps and two equal smaller cusps; (d) QAV with one larger cusp, two mid-sized cusps and one smaller cusp; (e) QAV with one larger cusp and three smaller cusps; (f) QAV with two equal-sized larger cusps and two unequal smaller cusps; (g) QAV with four unequal-sized cusps. In this study, QAV patients were categorized accordingly.

### Procedural details

All transcatheter aortic valve replacement procedures were performed with the patients under general anesthesia in the hybrid catheterization laboratory, and the preprocedural work-ups were completed according to institutional guidelines (15). CTA data were used for the accurate assessment of native aortic valve anatomy. Valve sizing was based on the area- or perimeter-derived mean diameter on CTA measurements by using the largest annular diameter in systole. The size of the transcatheter heart valve (THV) was selected based on a measured diameter of approximately 8–15% oversizing. For individual patients, balloon sizing could be additionally used for sizing the transcatheter prosthesis prior to THV deployment. Transapical or transfemoral approaches with a specific THV device were individually selected based on the dysfunctional status of the aortic valve for each patient.

### Transapical transcatheter aortic valve replacement

**FIGURE 2:**
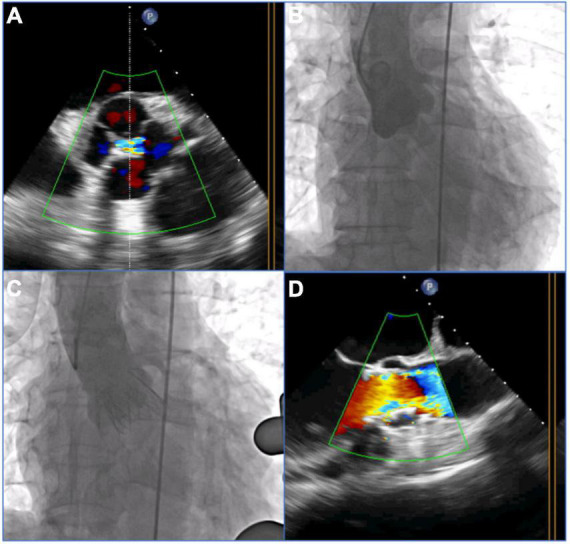
Procedural details of transcatheter aortic valve replacement (TAVR) with the self-expandable J-Valve prosthesis in a patient with a quadricuspid aortic valve with pure aortic regurgitation. **(A)** 2-Dimensional transesophageal echocardiography (TEE) with color Doppler showed aortic regurgitation in the quadricuspid aortic valve before the procedure. **(B)** Preoperative angiography showed four sinuses and aortic regurgitation. **(C)** Postoperative angiography showed the final position of a 29 mm J-Valve prosthesis without a paravalvular leak after deployment. **(D)** 2-dimensional TEE with color Doppler showed no aortic regurgitation postprocedure.

The patients with pure AR in the underlying population were treated by implanting the J-Valve prosthesis (Jiecheng, Suzhou, China) *via* a transapical approach. The J-Valve system is a self-expanding THV with a unique two-piece structural design consisting of three U-shaped graspers encircling the valve stent and has been certified by the China Food and Drug Administration for treatment of AR and AS. Previous publications described the detailed characteristics of the J-Valve system and its implantation process (16, 17). Briefly, after apical access was established *via* a left minithoracotomy, an apical puncture was performed, and a guidewire was advanced into the abdominal aorta *via* the apex. The J-Valve delivery system was then inserted into the ascending aorta. Three “U-shaped” claspers were released above the native aortic valve and then gently pulled down to three sinuses. For patients with QAV, three “U-shaped” claspers might have been pulled into three of the four sinuses. Aortic root angiography and transesophageal echocardiography (TEE) were used to evaluate the positions of the claspers. Once the claspers were in the correct positions, the valve positioning and deployment could be easily achieved by the guidance of the anatomically positioned claspers (Figure 2).

### Transfemoral transcatheter aortic valve replacement

**FIGURE 3:**
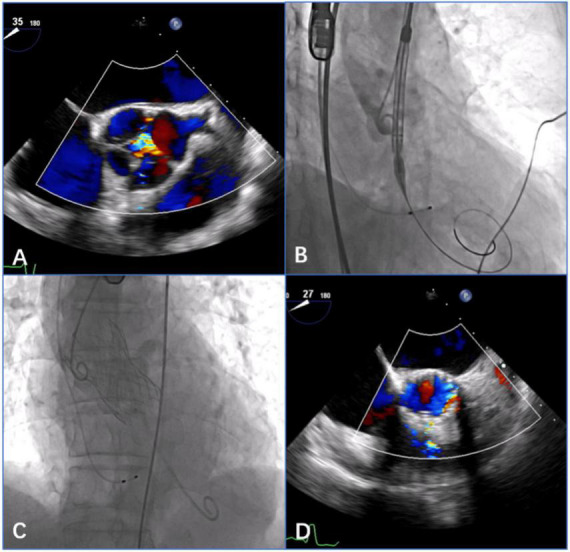
Procedural details of transcatheter aortic valve replacement (TAVR) with the self-expandable Venus-A prosthesis in a patient with quadricuspid aortic valve with aortic stenosis (AS) and regurgitation. **(A)** 2-Dimensional transesophageal echocardiography (TEE) with color Doppler showed aortic regurgitation in the quadricuspid aortic valve before the procedure. **(B)** Angiography showed a satisfactory position of the THV prosthesis before deployment. **(C)** Angiography showed the final position of a 32 mm Venus-A prosthesis without a paravalvular leak after deployment. **(D)** 2-Dimensional TEE with color Doppler showed no aortic regurgitation postprocedure.

The patient with AS in the underlying population was treated by implanting a Venus-A prosthesis (VenusMedtech, Hangzhou, China) via a transfemoral approach. The self-expandable Venus-A valve has a supra-annular design similar to that of the Medtronic CoreValve but a stronger radial force at the inflow end, which may be advantageous for patients with severe calcification. The procedure was performed via a transfemoral approach. The procedural details of TAVR with the self-expandable devices have been described previously (15, 18). Balloon predilatation is a routine procedure for patients with severe AS. Postdilatation and a valve-in-valve implant might be considered if moderate or severe AR appears after THV deployment (Figure 3).

### Statistical analyses

Statistical analyses were conducted with SPSS 22.0 software (IBM SPSS Statistics for Macintosh, Version 22.0. IBM Corp, Armonk, NY, USA). Continuous variables are presented as the means ± standard deviation, and categorical variables are expressed as percentages.

## Results

### Baseline characteristics

Five patients were identified from January 2016 and January 2022, with a mean age of 73.8 years (range 69–82 years); four patients were male. The echocardiography confirmed severe AR in four patients and severe AS and moderate regurgitation in the other patient. Preprocedural computed tomography (CT) scans revealed congenital QAV without severe dilation of the ascending aorta in all patients. Two patients had type B and the other three had type A, type D, or type F, respectively, according to the Hurwitz and Roberts classification for QAV. The mean score of the Society of Thoracic Surgeons for predicting the risk of mortality was 7.51% (range 2.668–18.138%). Baseline characteristics and comorbidities are listed in Table 1. Patients were recommended to be candidates for TAVR procedures by the heart team, and CT measurements suggested the feasibility of TAVR in all patients (Table 2). The patient with AS had mild calcification in the aortic leaflets. The aortic annular perimeters and their areas were 87.1 ± 5.1 mm and 595.5 ± 69.8 mm^2^, respectively. There was no risk of coronary ostia obstruction in any of the five patients. Coronary protection was not necessary based on CT measurements and *in vitro* simulation by a 3D printing model. The vascular diameters of the femoral access sites were greater than 7 mm in the transfemoral procedure, which is suitable for a 20 Fr sheath. Four patients with pure AR underwent transapical TAVR with the J-Valve system, and the patient with severe AS underwent transfemoral TAVR with the Venus-A system.

**TABLE 1:** Preoperative clinical characteristics.

Patients	Sex	Age (years)	Body weight (kg)	Functional status of aortic valve	Quadricuspid type (Hurwitz and Roberts classification)	Comorbidities	LVEF	NYHA FC	STS score
Patient 1	Male	82	61	AR	A	Hypertension	40%	IV	18.138%
Patient 2	Male	72	73	AR	F	Hypertension	38%	IV	4.055%
Patient 3	Female	71	58	AR	B	Hypertension	45%	III	4.380%
Patient 4	Male	69	72	AR	D	–	52%	III	2.668%
Patient 5	Male	75	52	AS-AR	B	Heart failure	33%	III	8.312%

AR, aortic regurgitation; AS, aortic stenosis; LVEF, left ventricular ejection fraction; NYHA FC, New York Heart Association functional class; STS, Society of Thoracic Surgeons.

**TABLE 2:** Preoperative computed tomography (CT) measurements.

Patients	Quadricuspid type (Hurwitz and Roberts classification)	Calcification	Annulus perimeter (mm)	Annulus area (mm^2^)	Sinus diameter (mm)	STJ diameter (mm)	LCA height (mm)	RCA height (mm)
Patient 1	Type A	No	84.3	555.7	LC 33.1 RC 36.6 NC 34.3 NC1 35.6	30.3	14.1	18.0
Patient 2	Type F	No	90.3	635.5	LC 33.6 RC 34.9 NC 38.8 NC1 33.4	29.5	13.3	13.6
Patient 3	Type B	No	80.2	500.5	LC 38.2 RC 35.9 NC 37.1 NC1 30.0	28.9	7.5	6.9
Patient 4	Type D	No	93.3	680.4	LC 39.1 RC 40.7 NC 38.1 NC1 37.3	33.0	8.7	18.5
Patient 5	Type B	Mild	87.5	605.3	LC 37.9 RC 37.1 NC 37.6 NC1 33.5	26.9	9.7	12.3

LC, left coronary; LCA, left coronary artery; NC, non-coronary; NC1, the other non-coronary; RC, right coronary; RCA, right coronary artery; STJ, sinotubular junction.

### Procedural outcomes

Procedural success was achieved in all patients, and the procedural characteristics are summarized in Table 3. Two patients received 29 mm J-Valve prostheses, two patients received 27 mm J-Valve prostheses, and the remaining patient with AS received a 32 mm Venus-A prosthesis. Preballoon dilatation was performed in one patient with severe AS. No postdilatation was required during the procedures, and there were no in-hospital deaths. Aortic root angiography and TEE were used to evaluate the position and function of the prosthesis. The proper positioning of the THV was confirmed in all patients. The mean pressure gradient decreased from 50.5 to 6.0 mmHg in the patient with AS. A trivial paravalvular leak was detected in only one patient postoperatively. TEE and aortic root angiography showed no AR and no paravalvular leak in any patient with AR treated with the J-Valve prosthesis.

**TABLE 3:** Procedural and postprocedural characteristics.

Patients	Approach	TAVR devices used	Procedural duration (min)	Fluoroscopic duration (min)	Predila -tation	Postdila -tation	Procedural success	Postprocedural aortic status	Procedural complications	Length of hospital stay (days)	In-hospital and 30-day complications	1-year LVEF	1-year NYHA FC class
Patient 1	Transapical	J-Valve 27 mm	130	15	No	No	Yes	Normal	No	9	No	51%	III
Patient 2	Transapical	J-Valve 29 mm	110	17	No	No	Yes	Normal	Hemorrhage, needed blood transfusion	5	No	48%	II
Patient 3	Transapical	J-Valve 27 mm	110	13	No	No	Yes	Normal	No	5	No	51%	I
Patient 4	Transapical	J-Valve 29 mm	100	15	No	No	Yes	Normal	No	4	No	54%	I
Patient 5	Transfemoral	Venus-A 32 mm	60	22	Yes	No	Yes	Normal	Trivial PVL	7	A-V block	45%	II

LVEF, left ventricular ejection fraction; NYHA FC, New York Heart Association functional class; PVL, paravalvular leak; TAVR, transcatheter aortic valve replacement.

All patients were extubated in the catheterization laboratory. One patient with AR received a blood transfusion because of a hemorrhage at the apex during the procedure. The patient with AS received a permanent pacemaker due to a high-degree atrioventricular block that occurred three days postoperatively. All patients recovered before discharge. There were no other major postprocedural complications, and the median length of hospital stay was 6 days.

### Follow-up

The median follow-up period was 18 (12–56) months, and the follow-up was 100% complete. After hospital discharge, no deaths occurred during the 30 day and 1 year follow-ups. All patients were alive at the time this manuscript was completed. All patients improved by ≥1 NYHA functional class at 30 days. Four patients were in NYHA functional class ≤II later in the follow-up period. All patients’ heart failure symptoms improved considerably. Two patients were readmitted to the hospital with non-specific chest discomfort within 1 year of follow-up; the results of all investigations were negative, and the patients were discharged home without further issues. The mean pressure gradients fell significantly in the patient with AS after the procedure and remained low throughout the follow-up period. No more recurrent regurgitation was observed in any patient during follow-up.

## Discussion

Compared with a regular tricuspid aortic valve (TAV), a QAV is associated with a significantly higher incidence of AR and stenosis over a lifetime. Surgical replacement or repair has been the standard treatment for QAV with pathological AS or AR in the past. With the rapid growth of the TAVR procedure, an increasing number of older patients with aortic valve disease are treated with this minimally invasive procedure, including those with QAV. Generally, CT scans are typically performed prior to TAVR for indication screening and strategy planning. CT evaluations can display the anatomical features of the aortic root and leaflets in detail. Therefore, the preoperative detection rate of QAV is currently higher than it was in the past. Blanke et al. (19) first documented the use of TAVR in patients with QAV in 2011. Blanke and his colleagues successfully implanted a 26 mm Edwards Sapien THV (Edwards Lifesciences, Irvine, CA, USA) via a transapical approach in a 79 year-old patient with AS-QAV. In the decade since, only a few successful cases of TAVR for the treatment of patients with QAV have been reported (Table 4) (10–14, 19–26). The pathological types of these patients included AS and AR. In accordance with the Hurwitz and Roberts classification, the reported cases covered types A, B, C, and E, and other cases did not express a specific valvular classification. Devices used in TAVR procedures included balloon-expandable valves, self-expandable valves, and novel self-expandable valves with positioning clips. Transapical or transfemoral approaches were used during TAVR procedures, depending on the selected devices, and the indication for the particular patient.

**TABLE 4:** Summary of the literature on quadricuspid aortic valve.

Study	Age, years	Sex	Functional status of aortic valve	QVA type (Hurwitz and Roberts classification)	TVAR device used	Approach	Postprocedural outcomes and complication	Follow-up performance	Follow-up duration
Zhou et al. (10)	79	Male	Severe AS and moderate AR	Type E	23 mm venus-A (Medtech)	Transfemoral	Normal	No major adverse cardiovascular events	5 years
	86	Male	Severe AS and moderate-to-severe AR	Type B	26 mm SAPIEN XT (Edwards lifesciences)	Transfemoral	Normal	No major adverse cardiovascular events	3 years
Han et al. (20)	70	Male	Symptomatic AS and AR	NA	26 mm venus-A (Medtech)	Transfemoral	Valve-in -valve because of severe PVL	NA	NA
Luo et al. (21)	62	Male	Severe AR and mild AS	Type A	27 mm J-valve (Jiecheng)	Transapical	Normal	No cardiac events	6 months
Fukui et al. (11)	74	Female	Severe AS with moderate AR	Type B	23 mm SAPIEN 3 (Edwards lifesciences)	Transfemoral	MPG decreased to 19 mmHg with mild PVL	No cardiac events	3 months
Takahashi et al. (22)	84	Female	Severe AS and AR	NA	23 mm SAPIEN 3 (Edwards lifesciences)	Transfemoral	Left main coronary ostia obstruction rescued with a stent deployed	NA	NA
Benkemoun et al. (12)	87	Female	Severe AS	Type A	23 mm Edwards SAPIEN 3	Transfemoral	Normal	No cardiac events	6 months
Aoyama et al. (13)	83	Male	Severe AS and AR	Type B	29 mm Evolut R (Medtronic)	Transfemoral	Trivial PVL	NA	NA
Tohoku et al. (14)	85	Female	Severe AS and moderate AR	Type C	23 mm SAPIEN 3 (Edwards lifesciences)	Transfemoral	Normal	NA	NA
Ibrahimet al. (23)	82	Female	Severe AS and moderate AR	NA	23 mm SAPIEN 3 (Edwards lifesciences)	Transfemoral	Normal	NA	NA
Sidharta et al. (24)	90	Male	Severe AS and moderate AR	NA	27 mm PORTICO (St. Jude medical)	Transfemoral	Trivial to mild AR	Significant symptom improvement	1 month
Bruschi et al. (25)	78	Male	Severe AS and moderate AR	NA	29 mm CoreValve (Medtronic)	Transfemoral	Normal	NA	NA
Yu and Lee (26)	80	Male	Severe AS and moderate AR	NA	26 mm SAPIEN XT (Edwards lifesciences)	Transfemoral	Normal	NA	NA
Blanke et al. (19)	79	Female	Severe AS and moderate AR	NA	26 mm Sapien (Edwards lifesciences)	Transapical	Normal	NA	NA

AR, aortic regurgitation; AS, aortic stenosis; NA, not available; PVL, paravalvular leak; QAV, quadricuspid aortic valve; TAVR, transcatheter aortic valve replacement.

Five cases of QAV were presented in this cohort. All patients successfully underwent treatment with TAVR. During this period, a total of 855 patients underwent TAVR procedures at our institution. Among them, 312 patients had AR. Therefore, the proportion of patients with QAV was estimated to be approximately 0.5% of the total cases and 1% of patients with pure regurgitation based on the data from our institution. Meanwhile, we retrospectively reviewed the clinical documents of patients undergoing aortic valve surgery during the same period at our institution. A total of 1,202 patients with aortic valvular diseases had operations, including six patients (0.5%) with QAV and regurgitation who underwent surgical aortic valve repair or replacement. Compared with patients who underwent TAVR, the patients who underwent open-heart surgery were relatively younger, with an average age of approximately 45 years. Therefore, the actual incidence of QAV may be higher than that reported previously in the literature, (1, 2, 5) and data obtained from autopsies and echocardiographic screenings alone may have limitations.

**FIGURE 4:**
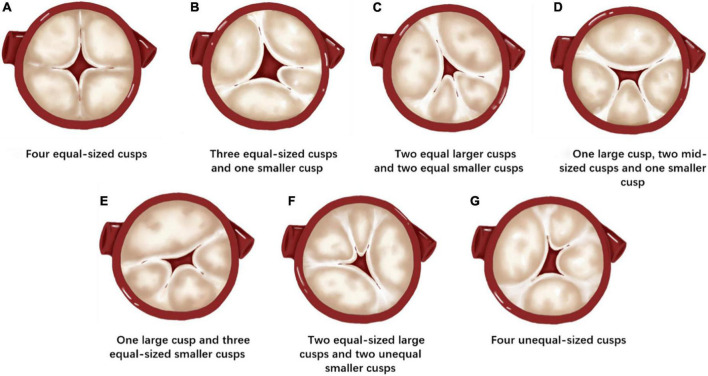
Hurwitz and Roberts classification of the quadricuspid aortic valve (QAV). **(A)** QAV with four equal-sized cusps. **(B)** QAV with three equal-sized cusps and one smaller cusp. **(C)** QAV with two equal larger cusps and two equal smaller cusps. **(D)** QAV with one larger cusp, two mid-sized cusps, and one smaller cusp. **(E)** QAV with one larger cusp and three equal-sized smaller cusps. **(F)** QAV with two equal-sized large cusps and two unequal smaller cusps. **(G)** QAV with four unequal-sized cusps.

Preoperative measurements and strategy planning for TAVR procedures are extremely important. The approach, type, and size of the THV, and procedural risk prediction are all based on preoperative measurements and evaluations. The Hurwitz and Roberts classification, (27) based on the relative size of the supernumerary cusp, divides QAVs into seven types from A to G (Figure 4). Because most patients with QAVs have undergone aortic valve replacement during surgery, (1, 5) this classification has limited significance for surgical guidance. For patients with QAV who underwent TAVR procedures, the preoperative assessment, and strategy planning were based on the current principles of routine TAVR assessment. However, certain specific anatomical issues of QAV may require more in-depth analysis. The TAVR strategy also needs to be adjusted based on these analyses to ensure better procedural outcomes.

Coronary ostia obstruction is a rare but life-threatening complication of TAVR. Persistent hypotension, typical ST-segment elevation, and ventricular fibrillation are common presentations of coronary ostial obstruction after THV deployment (28). Therefore, the risk of coronary ostia obstruction is an important issue in the preoperative evaluation of TAVR procedures. The risk factors for coronary ostia obstruction in patients having TAVR with regular TAV have been reported and include smaller sinus of Valsalva, low coronary height, and bulky valve leaflet calcification on CT scans (28–30). These principles can also be used to assess the risk of coronary ostia obstruction in QAV. However, there may be significant differences between QAV and TAV in the shape of the aortic sinus, the location of the coronary ostia, the length of a single valve leaflet, and the distribution of calcification. Patients with QAV may have different aortic sinus morphologies, including three or four sinuses. The volumes of individual aortic sinuses may vary considerably. The distribution of coronary ostia is also different from that of TAV according to the shape of the aortic sinus in QAV. Additionally, the lengths of the four leaflets in one patient may be different. These anatomical features in QAV may be related to the occurrence of intraoperative coronary occlusion. In Takahashi’s case report, (22) the routine preoperative assessment did not suggest a significant risk of coronary ostium occlusion with a 23 mm balloon-expandable THV. However, the left main coronary artery was occluded just after device expansion. One possible reason is that the QAV has a longer leaflet height and a shallower cusp depth compared with the TAV. Therefore, conventional pre-TAVR assessment methods in patients with TAV may not be fully suitable for assessing the risk of coronary occlusion in patients with QAV. Preoperative evaluation for TAVR in QAV should focus on the detailed measurement of sinus morphology, location of the coronary ostium, and the length of individual leaflets. For patients with a high risk of coronary artery occlusion assessed by CT, 3D printing and *in vitro* simulation may help to predict risk accurately and to develop procedural strategies.

Meanwhile, the selection of the size of the THV for QAV may also be different from that for TAV. The shallow cusp depth in QAV may result in a larger orifice of the aortic valve. According to the anchoring principle of the TAVR valve, a larger oversize may be needed. In Han’s case report, (20) a matching size of a self-expanding valve was selected according to the principle of conventional TAV evaluation. As a result, a second valve was implanted due to paravalvular leakage during the procedure. Postoperative analysis showed that a larger valve may be needed.

In addition, some TAVR devices are designed and manufactured specifically for patients with TAV, and their off-label use for patients with QAV requires preoperative safety and efficacy evaluations. Among the five patients with QAV treated with TAVR at our institution, four exhibited pure AR. The J-Valve system (JieCheng Medical Technology Co., Ltd., Suzhou, China) was used, and all cases achieved procedural success. Luo et al. (21) also reported a similar case with pure AR treated successfully with this device. The J-Valve system is a new generation of THVs with a unique design: a two-piece structure that consists of a three-prong clasper and a support frame. This design allows the THV to be implanted in two stages, which results in precise anatomical positioning and secure anchoring. Ideally, the three-positioning clasper should enter the individual aortic sinus accurately in TAV during the TAVR procedure, which ensures proper THV positioning afterward. Because QAV may have four aortic sinuses, it is difficult to enter the individual sinus with the positioning clasper during the procedure with the J-Valve. Even the THV can migrate after release due to blocking of the positioning clasper by the leaflet commissure. Therefore, preoperative assessment and prediction for this situation should be made if a device with a positioning clasper is used. At our institution, 3D printing models and preprocedural simulations are often used to predict outcomes and plan procedural strategies. The next generation of TAVR devices will have more design choices for positioning components, especially devices designed for the treatment of patients with pure AR. Positioning components may make the TAVR procedure more accurate and easier to perform. However, it is difficult to have commercially available devices with four positioning claspers specifically designed for QAVs because of their extremely low incidence. Therefore, attention should be given to matching the devices with three positioning components to the four leaflets and sinuses in QAV. In the future, devices should be selected carefully when this type of device is applied to QAV.

### Limitations

This study was done on a small cohort and represents early experiences at our institution. The relatively small number of patients did not allow us to make more convincing conclusion.

## Conclusion

Some patients with QAV develop AR or AS requiring surgical treatment at an older age. TAVR is an alternative treatment for this group of patients. Our early experience with TAVR in QAV demonstrates that these procedures are feasible with acceptable early results. The specific anatomical issues of QAV may require more in-depth analyses before procedures can be expected to ensure better outcomes. Further follow-up is necessary to determine the long-term outcomes of this modality.

## Data availability statement

The original contributions presented in this study are included in the article/supplementary material, further inquiries can be directed to the corresponding author.

## Ethics statement

The studies involving human participants were reviewed and approved by the Xijing Hospital Ethics Committee. The patients/participants provided their written informed consent to participate in this study. Written informed consent was obtained from the individual (s) for the publication of any potentially identifiable images or data included in this article.

## Author contributions

YL, MZ, and YM contributed to write and edit the manuscript. CX, LL, and YYM contributed to collect and edit the figures. PJ contributed to collect the data. JY contributed to the revision. All authors contributed to the article and approved the submitted version.

## Abbreviations

AR: aortic regurgitation
AS: aortic stenosis
CT: computed tomography
CTA: CT angiography
NYHA: New York Heart Association
QAV: quadricuspid aortic valve
TAV: tricuspid aortic valve
TAVR: transcatheter aortic valve replacement
3D: three-dimensional
THV: transcatheter heart valve.
